# Microbe Related Chemical Signalling and Its Application in Agriculture

**DOI:** 10.3390/ijms23168998

**Published:** 2022-08-12

**Authors:** Nur Wahida Abdul Hamid, Kalaivani Nadarajah

**Affiliations:** Department of Biological Sciences and Biotechnology, Faculty of Science and Technology, Universiti Kebangsaan Malaysia, Bangi 43600, Malaysia

**Keywords:** quorum sensing, quorum quenching, autoinducers, oligopeptides, N-acyl homoserine lactones, biofilm, extracellular polymeric substances, virulence

## Abstract

The agriculture sector has been put under tremendous strain by the world’s growing population. The use of fertilizers and pesticides in conventional farming has had a negative impact on the environment and human health. Sustainable agriculture attempts to maintain productivity, while protecting the environment and feeding the global population. The importance of soil-dwelling microbial populations in overcoming these issues cannot be overstated. Various processes such as rhizospheric competence, antibiosis, release of enzymes, and induction of systemic resistance in host plants are all used by microbes to influence plant-microbe interactions. These processes are largely founded on chemical signalling. Producing, releasing, detecting, and responding to chemicals are all part of chemical signalling. Different microbes released distinct sorts of chemical signal molecules which interacts with the environment and hosts. Microbial chemicals affect symbiosis, virulence, competence, conjugation, antibiotic production, motility, sporulation, and biofilm growth, to name a few. We present an in-depth overview of chemical signalling between bacteria-bacteria, bacteria-fungi, and plant-microbe and the diverse roles played by these compounds in plant microbe interactions. These compounds’ current and potential uses and significance in agriculture have been highlighted.

## 1. Introduction

**Figure 1:**
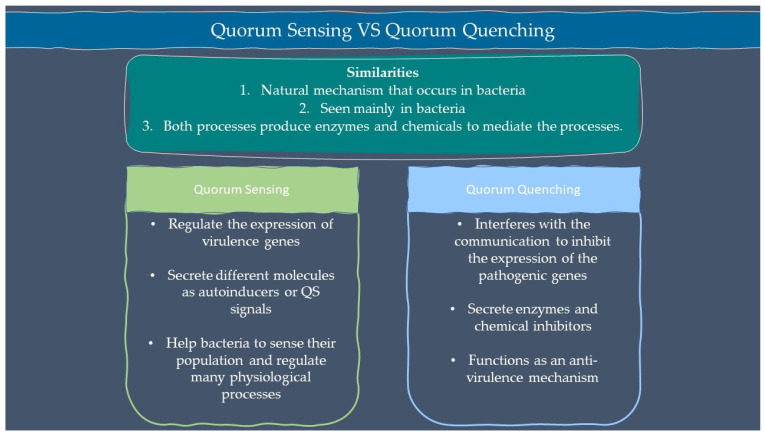
Summary of quorum sensing and quorum quenching.

Microbes are sensitive to the changes in their environment. In order to survive harsh environments, microbes alter their gene expression that affects microbial behavior. Microbes need to defend and protect themselves not only against the environment but also against other microbes that exist in the same niche. Communication is an important tool for all organisms to interact with each other. Microorganisms such as bacteria and fungi have a special way of interacting through chemical signal molecules known as autoinducers. These autoinducers trigger chemical communication between microbes. This communication process is called quorum sensing (QS), which allows bacteria and fungi to keep an eye on their surroundings for other bacteria/fungi and adjust their activity on a community scale in response to changes in quantity and species existing within a community. QS is important in microbes as it is used in the production of virulence factors, biofilm formation, and swarming motility [1,2]. Autoinducers are classified into three main types which are AI-1 (N-acyl homoserine lactones, AHLs), oligopeptides or autoinducing peptide and AI-2. Other than the above, there are a few more signalling molecules that are unique and do not belong to any classes such as diffusible signal factor (DSF), *Pseudomonas* quinolone signal (PQS), and diketopiperazine. AI-1 regulates Gram-negative bacteria QS while oligopeptides are discovered in Gram-positive bacteria. AI-2 is an interspecies autoinducer that is present in many species of Gram-negative and Gram-positive bacteria [3]. Quorum quenching (QQ) on the other hand is a process that interferes with quorum sensing. QQ is believed to have been developed as a natural method by QS-emitting species to clear their own QS signals, or competitive interaction with QS-emitters by QQ organisms. Furthermore, many different species employ QS to control the development and functioning of antimicrobials. Antimicrobial compounds are released by microbes to preserve the population stability which can cause injury or kill the target cells. Next, the capability to colonize a community is greatly influenced by the restricted amount of nutrients in the environment. Microorganisms with specialized metal acquisition systems such as siderophore can bind and promote the absorption of important metals from their environment, thus restricting the capacity of rival microbes to acquire necessary nutrients and able to colonize a community. A summary of quorum sensing and quorum quenching signaling is shown in Figure 1 below.

Current agricultural practices around the world depends on extensive use of chemical pesticides, herbicides and fertilizers which have a deleterious impact to the ecosystem and also human health. Awareness towards environmental sustainability has escalated the demand for organic products such as bioherbicide, biofertilizers and biofungicide. With the knowledge from QS, more organic products can be produced with adequate impact to the environment. Disruption of QS system can reduce significantly the virulence of phytopathogens. Moreover, knowledge from this QS system can be used to identify microbes that are antagonistic against phytopathogens and microbes that can be developed into commercial products. *Pseudomonas* sp., for example, are commonly utilized as biocontrol agents to tackle a variety of soil-borne infections, including *Fusarium oxysporum*, which causes *Fusarium* wilt. *Pseudomonas* sp. are able to inhibit the growth of other microbes with less potent siderophore. *Bacillus* sp., *Pseudomonas* sp., and various other Gram negative and positive bacteria have been reported to affect the soil environment as monocultures or as mixed cultures [4,5,6].

In recent years, a wide range of chemical signals produced by bacteria and fungi have attracted considerable interest in developing biofertilizer and biopesticides. To date, studies have contributed to significant progress in the knowledge of microorganisms’ communication mechanism. Understanding the process of chemical signaling among microbial populations has enabled researchers to recreate or regulate these interactions according to the suitability of a situation. For instance, experiment by Wubs et al., (2016) have demonstrated that transferring microorganisms that was found in a healthy soil to a dysbiosis soil over the period of six years can rehabilitate the soil health, thus improving the plant biodiversity and ecosystem [7]. Ultimately, the recent understanding of the involvement of advantageous microorganisms in agriculture and the understanding of host-associated microbial dysbiosis in a variety of situations have highlighted the need for techniques that can alter the structures and functions of host-associated microbial communities in agriculture.

Due to the recent advancements in omics technology and instrumentations, more chemical signaling compounds have been identified and characterized structurally and functionally for the role they play in plant-microbe interactions as well as microbe-microbe interactions [8]. Many of these chemical signals have been identified and developed into chemicals for use in agriculture such as Solvinix, Sarritor^®^ and Organosol [9,10]. In this review we look into both QS and QQ as well as various chemical signals that are produced by microbes in the environment, the roles played by these chemicals, the signaling involved, and how these chemicals have been, and may be used in the agricultural industry.

## 2. Communication Mode between Microorganisms

### 2.1. Quorum Sensing

Quorum Sensing is a communication systems used by microorganism, which is critical for the establishment of relationship between the microorganisms and their host [11]. QS is a social characteristic communication between bacteria and the environment in which bacteria creates and senses signal molecules to coordinate their behaviour in a population-dependent manner [1,2]. When QS molecules reach a certain level, bacteria adjust their gene expression pattern to cope with high cell density microbial cell surroundings. Unique extracellular signal molecules known as ‘autoinducers’ are associated with QS. N-acyl homoserine lactones (AHLs) are extensively studied autoinducer in Gram-negative bacteria, that possess an invariant lactone ring and acyl tail of varying lengths, saturations and presence of hydroxyl group [12]. These distinctions in its structure confers species uniqueness as well as differences in genetic regulation depending on the AHL receptor which serves as transcriptional regulator for a variety of bacterial community activities, including biofilm formation and pathogenicity [12].

The biofilm matrix is a harmonious community that helps to protect the microorganism from harsh environment and is vital for colonization [1]. Bacteria in biofilms are known to efficiently sustain communities by secreting extracellular chemicals that allow them to communicate with one another without having to come into direct physical contact. The LuxI is an autoinducer synthase enzyme that synthesizes AHLs, where the AHLs produced will interact with receptor proteins (LuxR homologues) in intracellular spaces of Gram-negative bacteria, and the dimers produced governing the phenotypic gene expression of biofilm formation, enzyme synthesis, manufacturing of antibiotics, and virulence factors [13]. Even at very low concentrations of AHLs, plants may detect their presence and respond in a variety of ways including changes in hormone levels involved in self-defence and the release of hormones associated with growth such as auxin, ethylene and jasmonic acid [14].

Oligopeptide autoinducers are used by Gram-positive bacteria as lead molecules. These autoinducing peptides (AIPs) are ribosomally produced and may have post-translational changes that affect the stability and functionality of their side chains [13]. Peptides typically need transporters to reach the extracellular environment, as they are impermeable to the bacterial membrane [15]. Diffusible signal factors (DSFs) are medium-chain unsaturated fatty acids that regulate QS in a variety of organisms, including *Burkholderia cenocepacia, Candida albicans*, *Pseudomonas aeruginosa*, *Stenotrophomonas maltophilia*, and *Xylella fastidiosa*, implying the involvement of inter-kingdom signalling pathways. *Cis*-2-dodecenoic acid, *cis*-11 methyldode-ca-2,5-dienoic acid, *cis*-11-methyl-2-dodecenoic acid, *cis*-10-methyl-2-dodecenoic acid, and trans-2-decenoic acid are examples of DSF compounds [12]. The first discovered DSF was *cis*-11-methyl-2-dodecenoic acid which was discovered in the *Xanthomonas campestris* pv. *campestris.* It influences the expression of extracellular enzymes such as Egl and protease, virulence factors and xanthan, as well as the regulation of pathogenicity factors (*rpf*) genes. The crotonase family enzyme rpfF, acts on fatty acyl carrier protein substrates, and the fatty acyl CoA ligase RpfB is required for *X. campestris* pv. campestris DSF production. A two-component system for DSF detection and signal transduction consists of the sensor RpfC, and the regulator RpfG [16,17]. Recognition of DSF by RpfC is related to phosphorylation of the HD-GYP which acts as the domain regulator and changes the cellular level of the second messenger cyclic di-GMP. Distinct pathways govern different subsets of Rpf-regulated virulence activities. RpfC favourably influences virulence factor production while adversely regulating DSF synthesis [17].

### 2.2. Quorum Quenching

Quorum quenching (QQ) is an interference to the QS system which will disrupt the attack of bacterial population. QQ possesses two main mechanisms; (1) QS signal molecule inhibitors (QSIs), and (2) QS signal molecule degradation enzymes. The QSI mechanism stops signal molecules from interacting with receptor proteins, thus interfering with QS, while the other mechanism reduces signal molecules by generating degrading enzymes, resulting in QQ [15]. Extracts of beans, clover, pea, garlic, geranium, grape, lily, lotus, pepper, strawberry, soybean, vanilla, and yam reduce AHL of QS in a variety of bacterial species [13]. Lactonase present in these plant extracts have QQ action. Lactones such as patulin and penicillic acid found in fungi behave as bacterial AHL signal counterparts. Patulin can be found in apples, pears, peaches, apricots, bananas, and pineapple, making these foods promising anti-QS phyto resources [16].

AHLs can be destroyed or changed by lactone hydrolysis, amidohydrolysis and oxidoreduction [18]. The activity of AHL acylase and AHL lactonase enzymes has been documented to cause AHL degradation that may be caused by multiple phylum members including Proteobacteria, Actinobacteria, and Firmicutes [9]. Furthermore, bacterial oxidoreductases, such as those produced by *Rhodococcus* sp., have the ability to actively alter AHL [13]. Lactonases that catalyse the hydrolysis of the ester bond to open the AHL ring are classified into several classes based on their folds. Phosphotriesterase-like lactonases are a common type of lactonase which requires two metal ions and a TIM barrel fold (triose-phosphate isomerase) for proper functionality. TIM barrel proteins are crucial because it is needed to support wide range of enzymatic activities [19]. AHL lactonases have been shown to successfully hydrolyzes a variety of lactones, including QS AHLs ranging from C4- to C12-homoserine lactone (HSL) [16], with or without C3 alteration.

**Figure 2:**
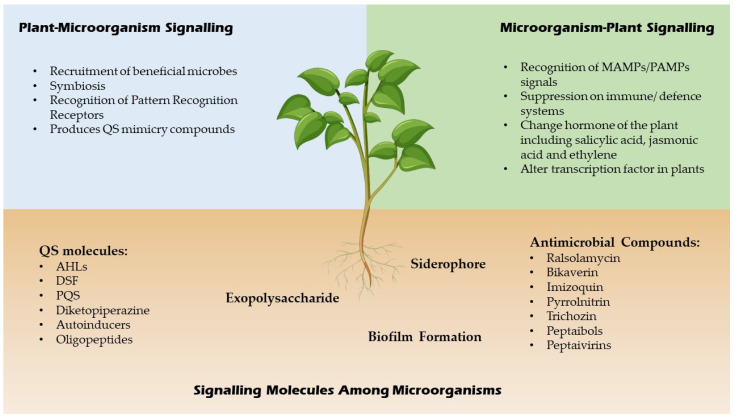
Signaling molecules of microbes and plants.

QSI are small molecules which have the capacity to effectively reduce quorum sensing controlled gene expression [20]. These compounds must be stable, specific and resistant to degradation as they will encounter different metabolic reactions in the cell. These compounds alter gene expressions of the targeted genes by binding to different promoters which may interrupt the interaction of the signalling molecules or prevent the synthesis of signal molecules hence inhibit the generation of secondary signals that modulate gene expression [20]. For example, a few *Bacillus* strains have been associated to *aiiiA* and *TasA* genes, which encode for many QSI, including lactonase, and have a broad spectrum antimicrobial action, suggesting that they might be used to manage bacterial diseases biologically [21]. In addition, furanones which are synthesized by fungi have a significant role as QSI for many Gram negative and positive bacteria by triggering the induction of stress response genes in a QS-independent manner [22]. A summary of signalling molecules produced by microbes and plants is shown in Figure 2 below.

### 2.3. Chemical Signalling in Fungi

One of the most prevalent chemical signaling molecule in fungi is farnesol. Following the discovery of farnesol in *Candida albicans*, it was discovered that lipids (oxylipins), peptides (pheromones), alcohols (tyrosol, farnesol, tryptophol, and 1-phenylethanol), acetaldehydes, and several volatile chemicals are actively engaged in fungal QS systems [20]. QS in fungi is often responsible for germination of spore, production of secondary metabolites, taxonomic transformation and enzyme secretions [23].

Intraspecies of fungi communicate with each other by releasing pheromones. Pheromones are used as signalling molecules to govern spore germination, production of secondary metabolites, structural transformation and enzyme secretion in fungi [23]. Pheromones produced are different based on the alleles expressed at the *MAT* locus [24]. For instance, *Saccharomyces cerevisiae* is one of the most popular and broadly described yeast where pheromones generated by this fungus cells are diffusible peptides which are known as a-factor and α-factor. Alleles expressed at the *MAT* locus will determine the peptide hormone and create only one of the two peptide pheromones. *MATα* is responsible for *α* expression where the pheromone precursor is encoded by *MFα1* that passes through numerous proteolytic processes before delivering a matured pheromone. *MATa* meanwhile is responsible for “a” expression, where the a-factor is farnesylated and can be recognized by ABC transporter Ste6p for a-factor secretion [24].

Mycoparasitic fungi such as *Trichoderma* sp. are commonly used in agriculture to combat other fungal pathogens such as *Rhizoctonia solani* and *Fusarium* sp. [25]. *Trichoderma* sp. produce a few metabolites including harzianopyridone, trichodermin and glivorin which have antifungal or antimicrobial properties that allow them to thrive in various environments [26]. *Fusarium* produces mycotoxins known as fusaric acid and deoxynivalenol (DON) which can activate defense mechanisms in *T. atroviride* and *Clonostachys rosea* which results in mycotoxin detoxification [27,28]. DON and fusaric acid also play an important role as a virulence factor that can cause *Fusarium* wilt in plant. DON synthesis is related to oxidative stress [29,30] while fusaric acid synthesis is related to metal ion content [31]. This two chemicals can hamper bacteria interaction by QQ of AHL in low concentration, and suppressing phenazine-1-carboxamide production at higher concentrations [32,33]. Other than that, zearalenone (ZEN) is another mycotoxin produced by *Fusarium* species. *C. rosea* however was reported to detoxify ZEN by breaking the ring structure of ZEN. *Trichoderma* sp. turns ZEN into sulphated form and reduces DON into its glycosylated form of deoxynivalenol-3-glucoside [27,34]. Table 1 below shows signal molecules produced by microorganisms and their respective functions.

## 3. Microbial Interactions and Chemical Signalling in Plant

### 3.1. Mycorrhizal Interactions

In many ecological niches, the coexistence of bacteria and fungus is a regular occurrence. The association of intracellular bacteria with their fungi inhabitants is considered to sustain ecological systems, in addition to being an important aspect of cellular evolution. The majority of known groups of fungus that include endosymbiotic bacteria are mycorrhizal fungi [47]. Bacteria can influence how fungi grow and evolve structurally. For instance, *Paenibacillus validus* secretes trisaccharide to induce hyphal and sporulation development of *Glomus intraradices* which enables AM fungus to complete its life cycle without a plant host [48] Other than that, it has been reported that *Rhizopus microsporus*, a pathogenic fungi which infects different crops including rice and maize, only sporulates when it is infected by *Burkholderia rhizoxinica* [49].

Apart from that, microbial communities frequently appear to exchange metabolites. Changing the availability of important nutrients may change the activity of the microbial companion. It has been demonstrated that certain fungus may induce a new phenotype in Streptomycetes by glucose deprivation which will allow colonization in different environments. This exploratory growth, uses a chemical mediator known as trimethylamine, to effectively transmit information to other actinomycetes [50]. The metabolites interchange might frequently be strictly controlled. For instance, mycorrhizal fungus *Laccaria bicolor*, secretes trehalose which acts as chemoattractant for *P. aeruginosa* and in exchange, the bacteria produces thiamine which will helps in the fungal development [51].

Apart from that, arbuscular mycorrhizal fungi (AMF) are also commonly found to establish mutualistic symbiosis with plant roots. AMF infiltrates the root system and it exchanges secondary metabolites which acts as nutrients between the host and the AMF [52,53]. Plants recruit microbes in the rhizosphere by releasing different exudates such as amino acids, hormones, sugars and nutrients that are beneficial for certain microbes and in exchange microbes release chemical that is beneficial for the plant [52]. For instance, biofilms of *B. subtilis* establishes a mutualistic relationship with the rhizome systems of the plant, allowing for pre-emptive colonization and preventing other pathogens from infecting the plant while allowing the bacteria to receive nutrients released by the plant roots [54]. Apart from that, AMF are also commonly found to establish mutualistic symbiosis with plant roots. Microbes in legumes rhizosphere are specific to their host and are recruited to the plant root system through chemical exudates released by the plant to recruit specific Rhizobia to form root nodules. This root nodule is important in legumes as it helps them in nitrogen fixation [55,56].

### 3.2. Nitrogen Fixation

Nitrogen is one of the most important elements in plant growth and development but plants cannot directly convert N_2_ in the atmosphere. In the rhizosphere, plants recruit bacteria, such as diazotrophs that are able to covert atmospheric nitrogen into a more useful form such as ammonia. *Rhizobium* spp., *Parasponia* spp., *Azospirillum* spp., *Frankia, Azoarcus* spp. and *Herbaspirillum* are few examples of diazotrophs [57]. Nitrogen concentration in the soil plays an important role in the diversity of nitrogen-fixing bacteria [58]. For instance, in the environments with low nitrogen, nitrogen-fixing bacteria that lives in root nodules will produce flavonols and flavones to entice and recruit legume-rhizobia symbiosis [58]. The flavones and flavonols stimulate the production of the bacterial *nod* gene, which starts the process of root nodulation. Inoculation of aerobic nitrogen-fixing bacteria into the rhizosphere of rice, wheat, and oat seedlings caused nitrogenase activity [57].

Nodulation process by rhizobia in the leguminous plants is a complicated and intriguing process which involves a number of biochemical interactions between the bacterium and its host [59]. During this interaction, bacteria are attracted to the plant roots by chemotaxis which causes root hairs to become curly. The formation of a nodule meristem is the result of the bacteria inducing cellular division in the typically dormant cells of the inner cortex of the root. An infection thread, which is a tube of plant origin that is created by bacteria trapped in the coiled root hair, is able to enters the exterior plant cells while the bacteria thrive within. 

*Frankia* produced different secondary metabolites including phenols, flavonoids and hydroxycinnamic acids where flavonoids have been shown to affect the diversity of the microbe community around it [57]. On the contrary, *Rhizobium* produced a unique signal molecules known as Nod factors which are essential for the uniqueness of the host-symbiont relationship as well as stimulation of all early plant responses, such as the transcription of symbiotic genes that causes the cortical cells to undergo mitosis again and the development of pre-infection threads [57,60]. On the other hand, plant is known to synthesized ethylene which is essential for plant growth and development. However, ethylene has a negative impact to nodulation [61]. Meddling with ethylene signaling increases nodule size and number. Contrary to the detrimental effect that ethylene has on nodule development, cytokinin mitotically reactivates cells in the pericycle and root cortex [62]. Additionally, isoflavonoids released by legumes including daidzein and genistein has a positive impact on *Bradyrhizobium japonicum nod* genes while *Sinorhizobium meliloti nod* genes was affected by luteolin. The degree of precision displayed helps the rhizobial community to precisely recognize their particular host.

### 3.3. PGPR Signalling

Microbes in the rhizosphere communicate predominantly through QS signalling molecules. At the reception of cognate signals, this cell-to-cell QS-based communication is implicated in plant growth promoting organisms colonization of plant roots, resulting in changes in gene expression corresponding to bacterial community density [63,64]. QS signalling molecules include antibiotics such as lipopeptide antibiotics that can be found in Gram-positive bacteria such as *Bacillus subtilis. B. subtilis* can be found easily in the soil’s top layers [54]. Biofilms of *B. subtilis* establish a mutualistic relationship with the rhizosphere of the plant, allowing for pre-emptive colonization and preventing other pathogens from infecting the plant while allowing the bacteria to receive nutrients released by the plant roots [54].

*Bacillus subtilis* is a plant growth promoting rhizobacteria (PGPR), that helps to solubilize phosphorus and enhance nutrient absorption. *B. subtilis* strains can synthesize lipopeptide antibiotics which can be divided into four groups which are the plipastatin, the surfactin group, the fengycin group, and the iturin group [65]. Lipopeptides are amphiphilic molecules with a low molecular weight. Lipopeptides genes are found in many bacterial species and strains of biocontrol agents, and some have been marketed for their improved ability to generate synthesized antibiotics and restrict root infections caused by fungi [66]. *Sfp* gene in bacilli is mandatory for a functionally active post-translational modification, which is required for synthetases of the non-ribosomal peptides. The *sfp* gene encodes a 4′-phosphopantetheinyl transferase that transforms inert apoenzyme peptide synthetases to their active holoenzyme forms post-translationally. A dysfunctional *sfp* gene will result in a lack of ability to synthesize antibiotics such as *B. subtilis* 168 which has a mutation in its *sfp* gene, but when it is complemented with a functional *sfp* gene, the antibiotic synthesis is restored [67]. Iturin has also been shown to disrupt yeast cell cytoplasmic membranes, resulting in the release of K^+^ ions and other essential elements, as well as the death of yeast cells [68]. 

Surfactin is a signal molecule (autoinducers) that activates the pathway involved in biofilm formation in *Bacillus* spp. [69]. Surfactin production is regulated by the srfA operon-*sfp* gene cluster system, and is crucial for cell differentiation. The *Sfp* gene is essential to build docking sites in the surfactin synthetase protein that allows particular amino acids to be loaded into the surfactin peptide chain and to activate the PCP domains by converting inactive forms to active units [70]. When surfactin is produced, potassium concentration in intracellular cell of *Bacillus* decreases due to the pore formation in the membrane. A sensor known as KinC will detect these changes and trigger SpoOA phosphorylation which then will stimulate the activation of genes that controls matrix production [71,72,73]. Further, when in contact with other species in the same ecosystem, surfactin produced by *Bacillus* will act as antibiotic by disintegrating cell membranes of other bacteria and fungi. For example, sulfate-reducing bacteria have been shown to be inhibited by surfactin produced by *Bacillus* sp. H2O-1 and *B. mojavensis* produced surfactin show antifungal activity against *F. verticilloides* [73,74]. A few *Bacillus* species are also known to synthesize fengycin groups which potentially suppresses filamentous fungi and inhibits phospholipase, a virulence factor in certain bacteria and fungi [75,76]. Membrane breakage, outflow of cellular contents, and eventual cell death of specific bacteria are all direct consequences of fengycin [75]. While fengycins have antifungal efficacy at low concentrations against a variety of fungi, their molecular processes are unknown and might vary depending on the pathogen’s target. In certain cases, this was clearly linked to spore/conidia permeability, which inhibits germination or, alternatively, causes hyphal cell disruption. Both behaviours are caused by CLPs degrading membranes, as seen by transmission electron microscopy. This action is most likely due to the compounds’ amphiphilic nature, which explains their strong affinity for lipid bilayers [77]. Aside from that, *Bacillus* sp. also produces bacteriocins, which are ribosomally synthesized short antimicrobial peptides that bacteria use to defend themselves against closely related bacterial species [78]. Bacteriocins are not only synthesized by Gram-positive bacteria but are also synthesised by Gram-negative bacteria and Archae. As a cationic peptide, bacteriocins can easily attach to the negatively charged phospholipid bilayers of the membranes and exert damage. Thuricin 17 is an example of a bacteriocin produced by Gram-positive bacteria whereas pyocin is synthesized by Gram-negative bacteria including *Pseudomonas* spp.

*P. aeruginosa* which can be found abundantly in the soil and water can synthesize bacteriocin known as pyocin [77]. Pyocin can be divided into three types: (1) R-type are non-flexible and contractile which resembles bacteriophage tails of *Myoviridae*, (2) F-type pyocin are flexible but non-contractile, and (3) S-type pyocin is a smaller protein compared to R and F-type pyocin with water soluble characteristics and very sensitive to heat protease [77,79]. The *prtN* activator regulates the expression of R-, F-, and S-type pyocin genes by binding to the P boxes of their promoters. In normal circumstances, *prtR* suppresses *prtN* expression. When subjected to stress conditions, an active RecA causes autoproteolytic cleavage of *prtR*, which results in the abolition of *prtN* repression and the synthesis of pyocin. A lysis cassette that encodes a holin (proteins that allow endolysin to pass through the cytoplasmic membrane by creating holes in the inner membrane) and an endolysin mediates the extracellular release of R-pyocin particles in *P. aeruginosa* [80,81]. *Pseudomonas* spp. can also produce biofilm, EPS and a few phenazine derivatives including pyocyanin, phenazine-1-carboxylic acid (PCA) and a few hydroxy-phenazines including 2-hydroxybenzoic acid which are also known as salicylic acid [82,83]. All of these chemical compounds act as signalling molecules and virulence factors for this genera [83].

*Trichoderma* sp. is a fungal mycoparasite that can recognize other fungi and inhibit their growth via a few modes of action. *Trichoderma* hyphae detect the presence of lectin on the antagonist fungi and secrete certain enzymes to degrade the cell wall of the targeted fungi [27]. To successfully parasitize the antagonist fungi, *Trichoderma* spp. excretes metabolites such as pachybasin, bisvertinolone and siderophore to help parasitize more efficiently [27,84]. Along with that, *Trichoderma* also produces pentenomycins, trichosetin, lignoren and cyclonerodiol that possess antimicrobial and antibiotic effects against Gram-positive and Gram-negative bacteria including *B. subtilis, Mycobacterium smegmatis* and *P. aeruginosa* [85]. Table 2 below shows other quorum sensing molecules produced by organisms that dwell in the rhizosphere.

Furthermore, Burkholderia are famous endophyte species found ubiquitously in the environment; including the *B. cenocepacia* and *B. tropica* that can be found in plant roots [91]. *B. cenocepacia* regulates bacterial pathogenicity through two different types of QS systems including AHL and the *cis*-2-dodecenoic acid (BDSF) system [92]. These two QS systems have combined effects on biofilm formation, virulence factor production, and bacterial motility in *B. cenocepacia* [93]. Study by Chen et al., (2020) has also shown that *B. cenocepacia* produced various volatile organic compounds (VOCs) such as indole, dimethyl trisulfide, allyl benzyl ether and methyl benzoate that have antifungal activity against different types of fungal pathogens including *Botrytis cinerea, Alternaria alternata* and *Bipolaris sorokiniana* [93]. Other authors also have reported similar results using different *Burkholderia* spp. which can hamper conidial germination of *B. cinerea* [94]. Further, research carried out by Tenorio-Salgado et al., (2013) shows that hyphal morphology of *F. culmorum* and *F. oxysporum* changed in the presence of *B. tropica* which eventually led to the death of the fungi [95].

### 3.4. Siderophore

Iron is also one of the important elements to all living organisms for numerous enzyme activities. In spite of that, iron is limited to the plant microhabitat and to survive this, endophytes should be endowed with features that facilitate its acquisition. Gram-negative bacteria such as *B. phytofirmans, G. diazotrophicus* and *Enterobacter* sp., have special traits that can synthesize and excrete low molecular weight molecules with high and specific affinity for iron which are also known as siderophores [96,97]. Low molecular weight siderophores synthesized by PGPR can solubilize and sequester iron from the soil and then provide it to the plant cells. PGPR which have this special trait release siderophores into the environment to bind iron (III) and adsorb ferric-siderophore complexes through Ton-B-dependent outer membrane receptors [98]. Although not all PGPR have these unique characteristics, PGPR such as *Pseudomonas* sp. exploit siderophores synthesized by other microbes known as xenosiderophores as a source of iron [99]. For instance, *P. putida* use other *Pseudomonas* sp. siderophores to obtain iron for themselves. To obtain pyoverdines which was synthesized and secreted by other *Pseudomonas* species, *P.putida* needs PupB, an outer membrane receptor that is triggered by the presence of the pyoverdines. This signaling process requires three different proteins which are PupB receptor, PupR anti-sigma factor, and the PupI ECF sigma factor [89]. PvdA enzyme is involved in this biosynthetic pathway while PvdQ enzyme is involved in maturation of the pyoverdine. Apart from that, *Pseudomonas* also produced siderophore pyochelin which also have roles in virulence and EPS formation [89].

### 3.5. Endophytic Signalling

Endophytic microorganisms invade plant tissues without producing any obvious detrimental consequences [100]. Endophytes can be found in the phyllosphere and rhizosphere and adopt lifestyle that commonly start as epiphytes on the plant surface and gradually change to endophytes by invading the plant tissues. Endophytes use a variety of mechanisms to continuously accommodate changes in their surroundings, which are strictly regulated by plants. In order to maintain a stable relationship, endophytes create a number of compounds that assist plants develop and adapt to their surroundings [101].

Adhesion is one of the most important keys for epiphytic and endophytic microorganism colonization. Bacteria is able to attach to the plant due to the formation of biofilm composed of water, polysaccharides, extracellular DNA, RNA, proteins and ions [102]. The endophyte’s QS system is similar to other bacteria where autoinducers and peptides are used for communication. For example, AHL based systems which are more commonly found in Gram-negative bacteria were detected in *Burkholderia phytofirmans*, *Microbacterium populi*, *G. diazotrophicus*, *Burkholderia cenocepacia*, *Pseudomonas* sp. and *Nitromonas* sp., whereas autoinducer-2 system which were used by both Gram-positive and Gram-negative bacteria as interspecies communication, was identified in Enterobacter sp. [103].

*P. syringae* start their life as epiphyte and gradually change to endophyte when it has successfully invaded the plant tissue and caused necrotic spots which are indicators that the disease has started. *P. syringae* produced two types of EPSs and extracellular DNA which are alginate and levan [104]. Alginate is composed of copolymer of o-acetylated β-1,4-linked D-mannuronic acid and L-glucuronic acid [105]. AlgU are sigma factors that control gene expression associated to alginate biosynthesis enzymes such as *algD* gene that controls the type III secretion system (TTSS) effector expression which plays a significant role in virulence regulation by suppressing the plant defense. Furthermore, AlgU appears to be able to control the synthesis of coronatine (COR), that contributes to virulence by reducing stomatal-based defense in the early stages of infection and also in the development of biofilm [106].

### 3.6. Parasitism Interaction

#### 3.6.1. Diffusible Signal Factor (DSF)

DSF is one of the most important QS molecules in bacteria. It is a *cis*-11-methyl-2-dodecenoic acid which requires the *rpf* gene cluster to regulate pathogenicity [107]. A number of bacterial activities, such as pathogenicity, biofilm formation, motility, interaction with insect vectors, and antibiotic resistance, are influenced by signaling mediated by DSF family components [108]. The ability to interfere with DSF signaling may open up new possibilities for the management of bacterial infection. As mentioned above, DSF are encoded by the *rpf* gene cluster such as *rpfABCDEFG* genes which are involved in the generation of extracellular polysaccharides or exopolysaccharides (EPS) and extracellular enzymes. RpfC and RpfG form a system to detect and transform DSF signal, while RpfF is a crucial enzyme for the synthesis DSF [109]. Comprehensive research of DSF-mediated QS has been carried out on *Xanthomonas campestris* where it used *cis*-11-methyl-2-dodecenoic acid (DSF) to synthesize a yellow pigment called xantomonadin that aids in epiphytic survival and pathogenicity by acting as a barrier against ultra violet (UV) light [110]. *Xanthomonas oryzae* pv. *Oryzae* synthesized three different chemical signal molecules which are DSF (*cis*-11-methyl-2-dodecenoic acid), BDSF (*cis*-2-dodecenoic acid) and CDSF (*cis*-11-methyldodeca-2,5-dienoic acid). On the other hand, *X. axonopodis* synthesized DSF which is butyrolactones. This QS systems controls exopolysaccharides and xanthan production which is an important element for biofilm production and virulence in this species [111]. Biofilm formed by bacteria can protect them from diffusion of antimicrobial and antibiotic [112,113]. Additionally, Malamud et al., (2011) revealed that *X. axonopodis* used DSF to control both sliding and swimming motility which is crucial during several stages of biofilm formation including surface adhesion, maturity and dispersal [114].

Although at first DSF signaling was formerly believed to only be present in *Xanthomonas* spp., it was later found in several unrelated species including *Burkholderia cenocepacia*, *B. vietnamiensis*, *B. dolosa*, and *B. ambifaria* synthesized *cis*-2-dodecenoic acid also known as BDSF, while *Xylella fastidiosa* synthesized *cis*-2-tetradecenoic acid and *cis*-2-hexadecenoic acid also known as XfDSF and XfDSF2, respectively. Although *Pseudomonas aeruginosa* is able to detect the presence of these molecules through bacterial behaviourial changes, it is unable to synthesize DSF and BDSF, but was able to synthesize *cis*-2-decenoic acid instead [108,115].

#### 3.6.2. Exopolysaccharide (EPS)

Exopolysaccharides (EPS) are water soluble polymers which predominantly consist of carbohydrates and proteins, released by microorganisms and have a variety of biological functions, including as cell-to-cell communication, adhesion to surfaces, and defense [116,117]. EPS also plays an important role in biofilms formation. EPS role is to increase the biofilm community’s capacity to scavenge moisture and nutrients from the environment when either is scarce, encouraging sustained metabolism under unusual circumstances [118]. As a generic physical barrier, EPSs act as a safeguard to the microbes. Synthesis of EPSs are directly influenced by certain environmental pressures including moisture, temperature, acidity, and light intensity [119]. EPS produced by microbes serve a key role in adhesion to plant surfaces by producing biosurfactants to promote cuticular penetration, allowing the microbes such as bacteria to colonize the plant’s surface [112]. The EPS also aids in maintaining a hydrated layer around the bacteria and therefore protects them from desiccation. *Ralstonia* *solanacearum* is a Gram-negative plant pathogenic bacterium that has caused vascular wilt in crops and significantly reduced crop yield. *R. solanacearum* can be considered as epiphyte or endophyte as it can cause disease either by invading the plant tissue or by staying on the surface of the plants [120]. These bacteria can produce EPS and cause disease in plant. The build-up of EPS will interrupt water movement in plant vessels which ultimately will cause acute withering symptoms in infected plants [121].

Besides that, beneficial bacteria including *Azoarcus*, *Rhizobium*, *Azospirillum*, *Sinorhizobium*, *Burkholderia*, and *Bradyrhizobium* are also able to synthesize EPS [102]. The *gumD* gene in *Gluconacetobacter diazotrophicus* is essential for EPS biosynthesis, while *wssD* gene in *Herbaspirillum rubrisubalbicans* is responsible for cellulose production, where the inactivation of these genes will limit the bacteria adhesion to the plant surface [102,122]. Lipopolysaccharide, capsule polysaccharide, gel-forming polysaccharide, and glucans are just a few of the polymeric compounds that exopolysaccharide-producing rhizobia strains such as *R. legumi-nosarum* bv. *Trifolii* and *Rhizobium alamii* generate. These compounds are essential for the advancement of efficient symbiotic interactions between the host and bacteria and encourages nitrogen-fixing nods [123]. Further, *Ensifer meliloti* a diazotrophic bacteria was able to produce EPS such as succinoglycan which is required for development of root nodules. Mutants strains are that are unable to produce this EPS will develop nodules without any bacteria in it [59].

#### 3.6.3. Antimicrobial Compounds

Microbes employ antagonistic tactics to preserve population stability, such as the release of secondary compounds that can injure or kill the target cells. These metabolites might not, however, be produced in sufficient quantities to have deleterious consequences. The microorganisms that secrete these chemicals must also produce a lethal dosage that is substantial enough to be effective while reducing subsequent self-exposure to toxic levels that might be harmful. Antimicrobials are release only happens when a certain threshold of antimicrobial-producing cells is reached, which is made possible with the use of QS. Therefore, it is not surprising that many different species employ QS to control the development and functioning of antimicrobials [124,125].

For instance, *R. solanacearum* produces ralsolamycin, a lipopeptide that can enhance chlamydospore formation and Mucoromycota, Ascomycota, and Basidiomycota fungus, including *Fusarium fujikuroi* produce the antimicrobial bikaverin [53]. Ralsolamycin, also known as ralstonin A, is made by a biosynthetic gene cluster called PKS-NRPS [hybrid non-ribosomal peptide synthetase/non-ribosomal peptide synthetase/(rmy)]. Spraker et al., (2018) found that ralsolamycin altered the metabolic profile of *F. fujikuroi*, resulting in the production of not just bikaverin but also additional compounds, including the bioactive metabolite beauvericin. In this experiment, both metabolites show promising results in controlling *R. solanacearum*, suggesting that these metabolites may protect *F. fujikuroi* against bacterial invasion [126]. According to Khalid et al., (2018) however, ralsolamycin produced by *R. solanacearum*, can suppress *imqK* gene cluster in *Aspergillus flavus*, and produce imizoquin which helps promote germination of fungal spores and in turn reduce *R. solanacearum* population [127]. Due to contradictory information reported, further studies need to be conducted to determine the function of the ralsolamycin produced by the *R. solanacearum.* Apart from the above, pyrrolnitrin is yet another metabolite synthesized by different bacteria species including *Pseudomonas*, *Burkholderia*, *Cystobacter*, *Serratia* and *Enterobacter*, with antibiotic activity against different fungi species and bacteria [128]. Pyrrolnitrin produced have an excellent antifungal activity against plant pathogenic fungi such as *Phytophthora capsici* which caused blight and fruit rot and *Rhizoctonia solani* which caused wilting and stunting in many commercial crops around the world [129]. Pyrrolnitrin interferes with glycerol kinase which will lead to glycerol build up in cells and hence cause leaky cell membrane [128]. Among fungi, *Trichoderma* is the most famous bioagent against plant pathogenic microbes. *Trichoderma* sp. synthesize different signaling compounds that also act as antimicrobial such as trichorzin, peptaibols and peptaivirins. These compounds have also exhibited antiviral activity against *cucumber mosaic virus* and *tobacco mosaic virus* [121]. 

## 4. Chemical Signals in Plant-Microbe/Pathogen Interactions

Signals produced by both the host (plant) and the colonizers are used to communicate between plants and soil microorganisms. Plant roots produce exudates/mucilage which secretes molecules such as amino acids, cutin monomers, flavonoids, hormones, organic acids and sugars that play a huge role in microorganism diversity and microbial colonization in the rhizosphere. In plant-microbe communication, microbes create a variety of signalling molecules such as phytohormones (auxin, cytokinin, and gibberellins), which are involved in the direct control of plant growth and development. Microbes produce signals made up from carbohydrate and protein which are essential for them to survive. These are known as Microbe or Pathogen-Associated Molecular Patterns (MAMPs or PAMPs). Microbial components including chitosan, glycoproteins, peptidoglycan, chitin, LPS and flagellin are examples of MAMPs/PAMPs detected by plants [64]. MAMPs trigger a local basal immune defence in the roots, which can then be translated into systemic defensive responses mediated by regulatory networks that include salicylic acid, jasmonic acid, and ethylene signalling. Further, plants can produce quorum sensing mimic chemicals, which can interfere with bacterial quorum sensing. Table 3 shows examples of chemicals produced by plants which are components of QS mimicry and their effects. Homoserine lactone (HSLs) as described earlier is an important metabolite for Gram negative bacteria interactions. HSLs also play a significant role in plant immunity as reported by Schuheggerr et al. (2006) [130]. In an experiment where tomato plants were inoculated with an HSL-producing bacteria, *Serratia liquefaciens* MG1, the plant showed significant increase of systemic resistance towards *Alternaria alternata*.

Plants use MAMPs or PAMPs to distinguish between beneficial and harmful microorganisms. Different plasma membrane-localized Pattern Recognition Receptors (PRRs) that bind MAMPs and PAMPs and control plant immune responses have emerged in plants. RLKs (receptor-like kinases) are transmembrane PRRs with extracellular domains that are involved in detecting ligands and transmitting information from external stimuli. The RLK-mediated signal transmission of pathogen defence is influenced by elicitors, pathogens, and signal molecules produced during biotic responses. RLK responses are frequently influenced by particular ligands and pathogens [130].

Plants respond to PAMPs by triggering a defence response known as PAMP-Triggered Immunity (PTI) or MAMP-Triggered Immunity (MTI), the first line of defence that restricts pathogen colonization and prevents proliferation in most plant species, resulting in changes to plant cells such as callose deposition, stomatal closure, and ethylene induction. Pathogens, on the other hand, have discovered strategies to escape PTI signalling or avoid detection by the host through effectors such as the MiSSP7 protein, which is an important component of pathogenesis [106]. Plants have evolved resistance (R) genes that express the Nucleotide-Binding Leucine-Rich Repeat (NB-LRR) protein, allowing them to identify some of these effectors directly or indirectly. The recognition of a pathogen’s avirulence protein sets off a cascade of immune responses known as Effector-Triggered Immunity (ETI). During ETI, the defence signalling pathways are stimulated, including the salicylic acid (SA) and jasmonic acid (JA) pathways [136].

After the plant recognizes microbes via MAMPs and PAMPs, the defence mechanism is extended across the entire plant via plant chemical signalling such as salicylic acid (SA), jasmonic acid (JA) and azelaic acid (AzA). Plants also use defence hormones to regulate the expression of specific sets of defence genes. Two important mechanisms involved in the regulation of these defence genes are the JA and SA pathways. SA is synthesized within the cytoplasm and the synthesis was enhanced by defence inducing compounds such as benzothiadiazole (BTH) [64]. Increased SA levels alter cytoplasmic redox, causing disulphide bonds to be cleaved in *NPR1* (Non-Expressor PR1) oligomers, which controls their transport to the nucleus. Following the translocation of NPR1 monomers from cytosol into the nucleus, it works as a co-transcription factor with TGA transcription factor (TF) and activates genes involved in defence [64,137]. SA signalling pathway is important for plant defence mechanism by inducing resistance to infection against hemi-biotrophic and biotrophic pathogens while JA is primarily involved in modulating disease resistance against necrotrophic pathogens [138]. Both signalling molecules contribute differently to plant defence relying on the type of invasive pathogen [139]. Although cross-talk between these two distinct signalling pathways have shown synergistic response in certain environment, most cases have shown antagonistic response [139]. For instance, several *Pseudomonas* species and strain are able to suppress SA signalling pathway by producing secondary metabolites such as coronatine (COR) which enhance susceptibility of the host. COR produced by *P. syringae* can activate JA pathway in plants by mimicking jasmonyl-isoleucine (JA-Ile), a bioactive form of the plant hormone JA [64]. Suppression of SA signalling pathway in plants will increase the chance of hemi-biotrophic and biotrophic pathogens to successfully invade plants by inhibit immune response of the host [140].

## 5. The Success of Microbial Chemicals in Improving Crop Yield and Growth

Chemicals can alter the microbial community in the soil, change soil pH, pollute water from nutrient leaching and increase greenhouse gas emissions. Most of these chemicals are used either to give extra nutrients to the plants for better growth or to control pests or pathogens. Microorganisms produce a lot of secondary metabolites that can be used to help increase agricultural yield. Metabolites synthesized by microorganisms can be used in agriculture to inhibit disease or improve plant development. For example, a few PGPR species secrete phytohormones including auxin, gibberellic acids and cytokinins for communication and enhancement of plant growth and development [138].

Biofertilizers are the best alternatives for chemical fertilizers which include living microbes. PGPR are commonly used in biofertilizers because they can encourage plant growth and development by secreting different secondary metabolites to help plant absorb nutrients more efficiently or to help plant defence mechanisms. In non-legume crops such as wheat, barley, oat, rice, sunflower, maize, line, beetroot, tobacco, tea, coffee, and coconuts, certain organisms, such as Azotobacter, are popular biofertilizers that play a significant part in nitrogen fixation. Furthermore, the Rhizobiaceae family, which includes Rhizobium, Mesorhizobium, and Bradyrhizobium, produce siderophores, indoleacetic acid (IAA), and 1-aminocyclopropane-1-carboxylate (ACC) deaminase, which aids in the growth of legumes and delivers nitrogen to plants.

Bioherbicides are also used to control weeds by applying it directly to the targeted weeds to kill or inhibit their growth. For example, *Colletotrichum* and *Phytophtora* are commonly used to suppress agricultural weeds. One of the most successful bioherbicides is the tobacco mild green mosaic virus which is used to control weed known as tropical soda apple. Recently the use of phytopathogen as bioherbicide is receiving attention but more research should be carried out as it has risk to the commercially important crop plants. *P. syringae* pv. *tagetis* has shown promising results which causes up to 100% mortality against a few weed species [141]. There are a few genera of fungus that are often used as mycoherbicides, including *Phytophthora, Sclerotinia, Alternaria* and the most famous genera being *Colletotrichum* [142,143]. A few *Colletotrichum* spp. including *C. goleosporioides, C. higginsianum, C. orbiculare* and *C. truncatum* produce mycotoxin such as colletochlorin-A,-E and -F, orcinol, tyrosol and dirhamnolipid which targets weed such as *Aeschynomene virginica, Sonchus arvensis* and *Xanthium spinosum* [8,141,143,144,145,146].

Biofungicides are living organism-based pesticides that are used to control plant diseases caused by either fungi or bacteria that are sprayed on either phylloplane or rhizosphere. *Trichoderma* is one of the most famous genera that is currently used as a biocontrol agent. *T. harzianum* is the most well-known species from this genera that is used to combat diseases caused by soil-borne pathogens including *Rhizoctionia, Phythium* and *Fusarium* [147]. *Trichoderma* sp. reduces the number of pathogens via several ways, including competition for food where *Trichoderma* spp. grow faster and rapidly compared to the pathogen, excretion of chemical compounds that inhibit the growth of pathogens and it can grow in host plants as endophytes and support the growth of the host [148]. Other than that, fungi such as *C. albicans, Penicillium* spp. and *Aspergillus* spp. can produce chemical compounds known as farnesol, which may exhibit a potential as a new biofungicide. This compound can inhibit the growth of *R. solani* that causes detrimental plant diseases around the world by inducing apoptosis and disintegrating the cellular ultrastructure of the fungal hyphae [89]. Table 4 shows the lists of commercial biocontrol products which can be found in the market.

## 6. Genetically Modified Microbial Products in Agriculture

Chemical signaling between microbes and plants have shown to play a key role in the environment by carrying and delivering messages to ensure efficient communication between cells. One of the challenges in designing a new bio-agent is identifying beneficial microbes that have a higher potential of colonizing the environment and producing good results. Interactions between microbes and plants are very complex and require thorough knowledge to produce new products.

Natural products are a substance or chemical compound produced by microorganisms that can be found in the environment. On the other hand, natural product synthesis is an attempt to produce a complicated target molecules in order to produce a product that is analytically similar to the naturally existing compound. For product that contain living microbes, a specific strain that has proven beneficial and do not have any environmental or health risk will be identified for use. For example, the famous *Trichoderma* sp. are commonly produced via process known as solid state fermentation (SSF) [161,162]. SSF is a typical method for manufacturing metabolites including organic acids, biosurfactants and enzymes since it reduces agricultural waste and is both economical and environmentally friendly [161]. Every product will have a specific microbe strain that serves a specific purpose. For example, *T. harzianum N47* is used to enhance root growth of *Pisum sativum*, *T. Harzianum M10* is used to improve germination of tomato seeds and production of harzianic acid and *T. Harzianum SQR-T037* will be used to improve root growth in tomato and manufacture harzianolide [163,164,165]. Additionally, *P. fluorescens* strains CHA0 and F113 are used to manufacture indole acetic acid and *P. putida* strains WCS358 are used to produce pyoluteorin [166]. All these strains have undergone genomic modification to offer more effective strains and produce higher yield [166]. Modern technologies have allowed to create genetically modified organisms (GMO) easily in a shorter time. For instance, genome editing by using CRISPR (Clustered Regularly Interspaced Short Palindromic Repeats) has recently been considered as a method of generating new biocontrol in a relatively easy to use and accurate manner [167].

Moreover, genetic engineering has been used widely in agriculture industries to improve the quality and productivity of the crop. For instance, transgenic plants or also known as genetically modified plants used one or more genes from microbes have been introduced using recombinant DNA technology [168]. The gene used will provide that plant with a specific characteristic or quality. *B. thuringiensis* (Bt), for example, is the most significant insecticidal bacterium for controlling caterpillar pests, fly and mosquito larvae, and beetles [169]. Bt creates crystals made up of insect-toxic Cry and Cyt proteins that are water soluble and belong to the endotoxin class, which binds to and damages the cellular lining of insect digestive systems [170]. Bt also synthesizes vegetative insecticidal proteins (Vip) which are highly toxic to a few *Coleoptera* and *Lepidotera* species. Bt has been used in different types of commercial crops including corn, cotton, potato and soybean [169].

On top of that, *Trichoderma harzianum* endochitinase gene, *chit42* was used in transgenic tobacco and potato crop to prevent a few bacterial infections including *Botrytis cinerea, Alternaria alternata* and *Rhizoctonia solani.* In addition, the nutritional value of staple crops has been improved through genetic engineering in order to lower the mortality and morbidity rates associated with micronutrient malnutrition as well as to increase agricultural production, productivity, and wellbeing for the underprivileged populations in developing nations. For example, recently a biofortified rice line, Golden Rice 2E (GR2E) has been approved and declared safe for consumption by US Food and Drug Administration (FDA) [171]. This golden rice used *crt1* genes from *Pantoea ananatis* which helps to catalyze conversion of 15-*cis*-phytoene to all-trans-lycopene, hence boost provitamin A content [171]. Besides that, GR2E also has *pmi* gene from *Escherichia coli* strain K-12 which permits GR2E to convert mannose-6-phosphate into fructose-6-phosphate which can be utilized as a carbon source [171,172].

Next, microorganisms can develop new genetic features through mutations, which occur when a gene is altered accidentally (‘spontaneous mutation’) or intentionally (‘induced mutation’). Mutation also helps to produce more microbial products with lower costs by using the same amount of raw material. For example, *B. subtilis* RB14 was able to synthesize iturin A three times more than the wild type when the native promoter was replaced with *repU* promoter [173] and in another experiment, mutant *B. subtilis* THY-7 synthesized surfactin, 16 times more than the wild type by replacing P*srfA* promoter in native strain with P*groE* [174,175]. As mentioned before, the ability to form biofilm is one of the important traits for bacteria colonization. This ability is controlled by QS system in bacteria which needs specific genes. Deletion or mutation of these genes will effect biofilm formation, hence affect the virulence of the bacteria [176]. This knowledge can be applied to control virulence of phytopathogen. For example, mutation of gene *edpX1* which is responsible for biofilm formation and EPS production in *X. oryzae* will significantly reduce EPS formation while deletion of *dgcA* gene will significantly reduce biofilm formation [177]. Moreover, knockout mutants of the gene *fliM, pilX* and *epsF* in *Azocarpus* sp. affects the organism’s pathogenicity to rice root by reducing the efficiency of the bacterial motility and EPS production [84].

## 7. Conclusions

Microorganisms communicate in different ways to cope with harsh environments. AHL is one of the most well studied QS molecule in Gram-negative bacteria while oligopeptide is used mainly by Gram-positive bacteria. These chemical molecules are secreted by microorganisms either to protect themselves by building biofilm/EPS or to reduce the population of other species. Microbes from different parts of plants have their own unique ways of communicating and adapting to the different environmental stresses. Microbial chemical interactions- whether mediated by signalling, antagonism or competition for resources, is likely involved in the growth and development of plants. Although many of these advantageous interactions are well recognised, relatively little is understood about the signalling molecules that initiate these interactions or the signalling pathways that plants and soil creatures have developed to detect and react to these cues. Hence, understanding the role of each chemical produced by these microbes can help to develop new bio-agents for a better alternative to current synthetic pesticides and fertilizers. Although there are currently a lot of biofertilizers and biopesticides in the market, there are still a lot of pathogens that can only be eradicated by chemicals. Hence, continuous studies are required to help identify new candidates for bioagents and to help improve current bioagent quality. In addition, new technologies that propel this search for new bioagents is also welcomed to catapult this area of study further.

## Figures and Tables

**Table 1:** Signal Molecule Produced by Fungi and the Function.

Organism	Signal Molecule	Role	References
*Fusarium*	Fusaric acid	Virulence factor Reduces activity of *Pseudomonas* and *Bacillus*	[35,36]
*S. cerevisiae*	Tryptophol	Promotes pseudohyphal growth	[37]
	Farnesol	Induces cellular death Increases mitochondrial reactive oxygen species concentration	[38]
*Debaryomyces nepalensis*	Phenylethanol	Promotes biofilm formation	[39]
*Penicillium* spp. and *Aspergillus* spp.	Patulin	Virulence factor Fungal colonization Antimicrobial activity	[40,41]
	Oxylipins	Virulence factors Growth factors during sexual development Controls mycotoxin production	[42,43]
*Penicillium sclerotiorum*	Multicolic acid	Accelerates the process of sclerotiorin synthesis	[23]
*Fusarium culmorum*	Terpenes	Influences swimming and swarming motility of *Serratia* *plymuthica*	[44]
*Penicillium decumbens*	Farnesol	Promotes the growth of hyphae Increases the secretion of cellulase	[45]
*Penicillium expansum*	Farnesol	Induces cellular death Inhibits the growth of hyphae	[46]
*Fusarium graminearum*	Farnesol	Inhibits growth of hyphae	[46]

**Table 2:** Quorum Sensing Molecules Produced by Rhizospheric Microbes.

Organism Producing Quorum Sensing Molecule	Quorum Sensing Molecule Produced	Role of the Quorum Sensing Molecule	References
*Arthrobacter agilis*	Dimethylhexadecylamine	Affects bacterial growth and swarming motility of *Bacillus* and *P. fluorescens*	[86]
*B. licheniformis*	ComX pheromone	Inhibits the growth of *A. flavus*	[87]
*B. subtilis* subsp. *Subtilis* C9	Acetylbuanediol	Affect the growth *Rhizoctonia solani*	[88]
*P. aeruginosa*	Rhamnolipids	Act as biosurfactants to reduce tension on surfaces for motility	[89]
*P. fluorescens*	2,4-Diacetylphloroglucinol	Promote mycelial growth and root colonization of *Glomus mosseae*	[80]
*Pseudomonas* spp.	Dimethyl disulphide	Inhibit germination of *S. sclerotiorum*	[81]
*Sinorhizobium meliloti*	*N*-(tetrahydro-2-oxo-3-furanyl)-octanamide (C_8_-HL)	Biofilm formation Influence nodulation efficiency	[90]

**Table 3:** Plant quorum sensing mimicry molecules and their effect on microbes.

QS Mimicry Molecule	Plants	Affected Microbes	Role	References
**Rosmarinic acid**	*Rosmarinus officinalis, Salvia officinalis, Thymus vulgaris, Melissa officinalis, Symphytum officinale, Aegiphila mollis, Ocimum basilicum*	*P. aeruginosa*	Stimulates early QS-responsive gene expression, hence reduces pathogenicity	[131,132]
**Eugenol**	*Anethum graveolens, Syzygium aromaticum*	*Chromobacterium violaceum, P. aeruginosa*	Prevents the development of virulence factors such as violacein, elastase, and pyocyanin, as well as the formation of biofilms.	[133]
**Curcumin**	*Curcuma longa*	*P. aeruginosa*	Inhibits the expression of virulence genes	[134]
**Naringenin**	*Citrus* sp., *Ficus carica, Solanum lycopersicum*	*P. aeruginosa, C. violaceum*	Reduces the production of AHLs Inhibits the expression of virulence genes Inhibits production of pyocyanin and elastase	[134,135]

**Table 4:** Commercial biocontrol products.

Type	Marketing Name	Active Ingredients	Target Pathogen, Diseases or Weeds	Mode of Action	References
**Biofungicide**	AQ10 Bio Fungicide	Spores of a naturally occurring *Ampelomyces quisqualis* strain AQ10	Powdery mildew	Spores germinate into the powdery mildew mycelia and parasitize it	[149]
	Trichodex	*Trichoderma harzi anum* T39	*Botrytis cinerea* *Rhizoctonia* *Sclerotinia* *Colletotrichum* *Cladosporium fulvum*	Antibiosis Hyperparasitism Competition for nutrients and space Induction of resistance in the host plant Reduces pathogen spore dissemination capabilities	[150]
	Rootshield^®^ WP	*Trichoderma harzianum* strain T-22	*Pythium* *Fusarium* *Rhizoctonia* *Cylindrocladium* *Thielaviopsis* species	Mycoparasitism Competitive exclusion	[151]
	Binab T	*Trichoderma harzianum* and *Trichoderma polysporum*	*Botrytis* *Fusarium* *Pythium* *Phytophthora* *Rhizoctonia* *Verticillium*	Mycoparasitism Competitive exclusion	[151]
	Primastop	*Gliocladium catenulatum* Strain J 1446	*Rhizoctonia* *Pythium* *Phytophthora* *Fusarium* *Didymefla* *Botrytis* *Verticillium* *Alternaria* *Cladosporium* *Helminthosporium* *Penicillium* *Plicaria*	Produces hydrolytic enzymes including β-1,3-glucanase and chitinase Produces chemicals that inhibit the growth of fungi such as epipolythiodioxopiperazines, bisorbicillinoids, verticillin and peptaibiotics	[152]
	Contans WG	*Coniothyrium minitans*, strain CON/M/91-08	*Sclerotinia sclerotiorum*	Produces macrosphelide A to inhibit mycelium growth	[153]
	Biosave^®^	*Pseudomonas syringae* Strain ESC-11	Gray mold Mucor on pome fruits *Penicillium expansum* *Rhizopus stolonifer* *Fusanum sambucinum* *Helminthosporium solam*	Competitive inhibition Interruption of the metabolism of pathogenic organisms	[154]
**Bioherbicide**	Biochon	*Chondrostereum purpureum*	*Prunus serotina* *Populus euramericana*	Production of polygalacturonases and laccases lignin- and manganese peroxidase activities	[155]
	Dr. Biosedge	*Puccinia canaliculata*	Yellow nutsedge	Suppresses flowering Restricts new tuber formation	[156,157]
	Solvinix	Tobacco mild green mosaic tobamovirus (TMGMV)	*Solanum viarum*	Elicits the hypersensitive response of the weed	[158]
	Sarritor^®^	*Sclerotinia minor* IMI 344141	Dandelions	Inhibition of enzyme essential for amino acid production.	[159,160]
	Organo-sol	*Lactobacillus rhamnosus* strain LPT–21 *Lactobacillus casei* strain LPT–111 *Lactococcus lactis* ssp. cremoris strain M11/CSL *Lactococcus lactis* ssp. lactis strain LL64/CSL *Lactococcus lactis* ssp. lactis strain LL102/CSL.	Broadleaves weed	Production of lactic acid and citric acid that allows for penetration of plant cells and cause tissue necrosis	[160]
